# Blade-Coated Porous 3D Carbon Composite Electrodes Coupled with Multiscale Interfaces for Highly Sensitive All-Paper Pressure Sensors

**DOI:** 10.1007/s40820-024-01488-0

**Published:** 2024-08-13

**Authors:** Bowen Zheng, Ruisheng Guo, Xiaoqiang Dou, Yueqing Fu, Bingjun Yang, Xuqing Liu, Feng Zhou

**Affiliations:** 1 State Key Laboratory of Solidification Processing, Center of Advanced Lubrication and Seal Materials, School of Materials Science and Engineering, Northwestern Polytechnical University, Xi’an, 710072 People’s Republic of China; 2 Research Center of Resource Chemistry and Energy Materials, State Key Laboratory of Solid Lubrication, Lanzhou Institute of Chemical Physics, Chinese of Academy of Sciences, Lanzhou, 730000 People’s Republic of China; 3 State Key Laboratory of Solid Lubrication, Lanzhou Institute of Chemical Physics, Chinese Academy of Sciences, Lanzhou, 730000 People’s Republic of China; 4 Shandong Laboratory of Advanced Materials and Green Manufacturing at Yantai, Yantai, 264006 People’s Republic of China

**Keywords:** Micro- and nano-structures, PEDOT:PSS, Flexible pressure sensors, Health monitoring, Multiscale interfaces

## Abstract

Flexible and wearable pressure sensors hold immense promise for health monitoring, covering disease detection and postoperative rehabilitation. Developing pressure sensors with high sensitivity, wide detection range, and cost-effectiveness is paramount. By leveraging paper for its sustainability, biocompatibility, and inherent porous structure, herein, a solution-processed all-paper resistive pressure sensor is designed with outstanding performance. A ternary composite paste, comprising a compressible 3D carbon skeleton, conductive polymer poly(3,4-ethylenedioxythiophene):poly(styrenesulfonate), and cohesive carbon nanotubes, is blade-coated on paper and naturally dried to form the porous composite electrode with hierachical micro- and nano-structured surface. Combined with screen-printed Cu electrodes in submillimeter finger widths on rough paper, this creates a multiscale hierarchical contact interface between electrodes, significantly enhancing sensitivity (1014 kPa^−1^) and expanding the detection range (up to 300 kPa) of as-resulted all-paper pressure sensor with low detection limit and power consumption. Its versatility ranges from subtle wrist pulses, robust finger taps, to large-area spatial force detection, highlighting its intricate submillimeter-micrometer-nanometer hierarchical interface and nanometer porosity in the composite electrode. Ultimately, this all-paper resistive pressure sensor, with its superior sensing capabilities, large-scale fabrication potential, and cost-effectiveness, paves the way for next-generation wearable electronics, ushering in an era of advanced, sustainable technological solutions.

**Supplementary Information:**

The online version contains supplementary material available at 10.1007/s40820-024-01488-0.

## Highlights

- A blade-coated composite paste, composed of a compressible 3D carbon skeleton, PEDOT:PSS, and CNTs, can naturally dry to form a porous electrode on paper with a micro- and nano-structured surface.
- The all-paper pressure sensor demonstrated an ultrahigh sensitivity of 1014 kPa^−1^, a wide responsive range up to 300 kPa, and an ultralow operating voltage of 0.01 V.
- The sensor showcased superior detection capability, ranging from subtle wrist pulses and robust finger taps to large-area spatial force.

**Supplementary Information:**

The online version contains supplementary material available at 10.1007/s40820-024-01488-0.

## Graphical abstract

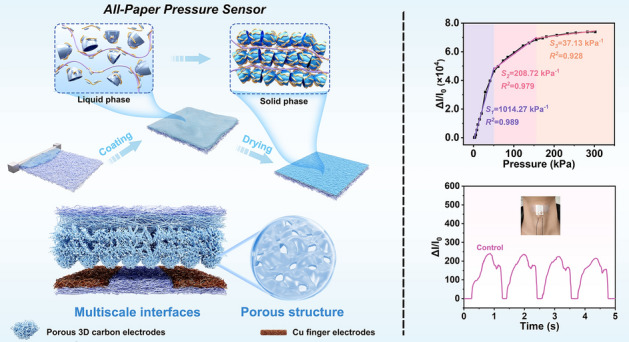

## Introduction

In an era where technology seamlessly integrates with daily life, the advent of flexible and wearable pressure sensors marks a significant milestone, especially in fields demanding precision and innovation, such as healthcare, robotics, and consumer electronics [1–6]. These sensors, celebrated for their versatility, are pivotal in advancing medical diagnostics, enhancing robotic functionalities, and enriching user interaction with electronic devices. The healthcare sector, in particular, has witnessed a transformative shift, embracing wearable technology that offers unprecedented convenience in monitoring and diagnosing, thereby redefining patient care paradigms.

However, the journey toward perfection is fraught with challenges. The pursuit of sensors boasting high sensitivity and an expansive response range often clashes with the practicalities of manufacturing complexities and cost constraints. In the case of piezoresistive pressure sensors, achieving high sensitivity typically involves enhancing material conductivity or designing structured sensing materials through morphological/structural engineering. The former relies on the material's electrical properties to induce resistance changes but has limitations in terms of sensitivity enhancement [1]. Conversely, the latter leverages the surface microstructures of elastic materials to initially alter sensor contact points, resulting in higher initial resistance and facilitating substantial resistance changes. Nonetheless, sensors utilizing these surface microstructures typically demonstrate sensitivities lower than 10 kPa^−1^ as well as a limited sensing range, which are fundamental requisites for tactile sensing in smart robotic manipulation or in gently manipulating objects [7]. Among the structured approaches, porous structures have been proposed to enhance sensing performance, whereby increased deformation capabilities result in higher sensitivity [8]. Interconnected porous materials, including foam, sponge, aerogel [9], paper, and textile-based network structures with adequate conductivity, have been developed. Nonetheless, these structures often exhibit compression contact saturation under high pressure, leading to a relatively narrow sensing range of less than 100 kPa. To further enhance the sensing performance of pressure sensors, a multiscale hierarchical structure strategy has been introduced to bolster the capabilities of piezoresistive pressure sensors. This includes an intrinsic hierarchical structure [10, 11], multilayer-stacked hierarchical structure [12], and hierarchical combined microstructure [1, 5, 13, 14]. Many multiscale hierarchical structures span from the nano to microscale. An innovative design leverages the mutual synergy across a nanoscale conductive film composed of carbon nanotubes (CNTs), a microscale spinous microstructure, and a milliscale arched-layer structure to optimize compression contact in pressure sensors [15]. This design demonstrates high sensitivity (15.1 kPa^−1^) and a wide detection range (180 kPa). Furthermore, a combined hierarchical structure with microrough and porous structures has been developed. This approach involves constructing a sensing layer with a double-sided pyramidal carbon foam array that integrates tapering microstructure and microscopic porosity, two prevalent means for enhancing sensitivity (24.6 kPa^−1^) and sensing range (ultra-wide linear range of 1.4 MPa) of piezoresistive materials [7]. Obviously, the structural strategy of this composite sensing material increases the deformable space and enhances the material's deformation ability, improving the material's sensitivity and broadening the sensing range [5]. Meanwhile, this strategy requires different microstructures to match each other well, which increases the difficulty of fabrication procedure for the composite sensing material. In short, various fabrication methods can meet the requirements for high sensitivity and wide sensing range, however, the demand for preparing sensitive materials remains relatively high, involving techniques such as photolithography, picosecond laser applications, high-temperature processes, vacuum deposition, and more. Consequently, there are still challenges associated with fabricating low-cost, high-performance, and large-scale pressure sensors.

To date, wearable pressure sensors used for detecting physiological signals have predominantly utilized ultra-thin substrates such as poly(ethylene terephthalate) (PET), polyamide, and silicone elastomers [16]. In contrast, paper, comprising cellulose fibers, is seen as an inexpensive, disposable, and biodegradable substrate material for wearable electronic products [17, 18]. Furthermore, it aligns with green electronics principles, emphasizing proper recycling and disposal of electronic waste to prevent environmental contamination and promote resource recovery. Consequently, paper finds wide application in various sensing fields, including biosensors, chemical sensors, and mechanical sensors for registering responses to mechanical stimuli, serving as an environmentally friendly and disposable product within the domain of green electronics. Most paper-based piezoresistive pressure sensors make use of microfibers and porous structures, enhancing sensing performance by coating thin films such as MXene [19–21], conductive polymer [22, 23], graphene [24–26], CNTs [27–29], Ag nanowires [30], and so on to form a composite sensing layer. However, these composite sensing materials often lack multiscale hierarchical structures, leading to unsatisfactory sensitivity and detection range, actually, reaching a pressure level of 300 kPa is essential to mimic human pressure perception [7]. Nonetheless, the existing composite sensing materials have relatively complicated fabrication process and are not suitable for mass production [31]. Therefore, to obtain multiscale hierarchical structures and simplify the material fabrication process, an ingenious composite sensing material should be developed as a more promising option.

As we all known, the conductive polymer poly (3,4-ethylenedioxythiophene)-poly(styrenesulfonate) (PEDOT:PSS) demonstrates outstanding electrical conductivity and can be processed into sensing materials using solution processing [32, 33]. Additionally, it is capable of forming compressible aerogels independently or in hybrid form with other carbonaceous materials through supercritical drying or freeze-drying methods [34, 35]. However, these drying techniques mentioned above can adversely affect the mechanical properties of cellulose paper, and thicker aerogels may impact wear comfort. Herein, we conceive a new strategy to develop high-sensitivity, wide-range, and cost-effective all-paper-based piezoresistive pressure sensors. Our previous work displayed that three-dimensional framework carbon (3DFC) can be dispersed in PEDOT:PSS aqueous solution [36]. The special 3DFC, with porous structure and its deformable single cell, is expected to act as a compressible sensing material when combined with conductive PEDOT:PSS. Better yet, 3DFC can be obtained by simply calcinating sodium citrate, making it feasible for high-throughput production [37]. Therefore, we employ PEDOT:PSS to uniformly coat the 3D carbon, forming a compressible composite that contains nanoscale porosity in its body and microscale waves with nanostructures on the paper surface, resulting a combined hierarchical structure. The 3D hierarchical electrode is created from an aqueous PEDOT:PSS-3D carbon paste, which is coated on paper using a simple blade-coating technique and naturally dried in an air environment. Simultaneously, to enhance sensitivity, a multiscale bottom metal electrode is designed on rough paper with micrometer fibers and porosity by screen-printing interdigitated electrodes with submillimeter widths. The top and bottom paper-based electrodes are stacked face to face, forming the all-paper pressure sensor, which features a submillimeter-micrometer-nanometer contact interface and nanometer porosity within the sensing layer body, i.e., a multiscale hierarchical structure. This constructed all-paper pressure sensor exhibits an ultra-high sensitivity of 1014 kPa^−1^ and a broad sensing range for applied pressures up to 300 kPa at a low voltage of 0.01 V. It is capable of real-time monitoring of human body-related signals such as pulse detection, human body acoustic signals, joint bending, and finger tapping. As a demonstration, a large-scale sensor array is printed as a recyclable and flexible sensing pad to detect the patient's hand grasping posture. This sensor, easy to fabricate and delivering exceptional performance, holds significant promise for diverse applications in wearable health-monitoring devices.

## Experimental Section

### Materials

PEDOT:PSS solution (Clevios PH1000, 1.2 wt%) was purchased from Heraeus. Sodium citrate and DMSO (99.7%) were purchased from Sinopharm. CNTs with hydroxyl were purchased from XF NANO, China. Ammonium tetrachloropalladate was purchased from Sigma-Aldrich. Qualitative filter paper (Jiaojie, China) was used as the top paper substrate, and PP membrane filter with 0.22 μm in porous diameter from commercial products (Hainingyibo, China) was used as the flexible substrates to carry Cu electrode. Copper wires were commercial products.

### Fabrication of All-Paper Pressure Sensors

#### Fabrication of the Interdigitated Electrodes

Copper interdigitated electrodes were screen-paper on filter paper as bottom electrodes using a previously reported matrix-assisted catalytic printing (MACP) method [38]. In particular, catalytic ink (23 mg ammonium tetrachloropalladate, 5 g polyethylene glycol, and 2.3 g deionized water) was poured onto a patterned screen and spread onto filter paper with a blade. The filter paper with the ink pattern was immersed in the electroless deposition solution to deposit the Cu pattern at room temperature.

#### Fabrication of the PEDOT:PSS@3DFC-CNT Top Electrodes

3DFCs were prepared via a direct calcination of sodium citrate at 700 °C reported by our previous work [37]. We prepared the mixture of 3DFC and CNT, which were mixed at a mass ratio of 1:1, 1:0.5, 1:0.33, 1:0.25, and 1:0. Finally, PEDOT:PSS aqueous solution containing 5 vol% DMSO was added and mixed in a mortar until the resulting solution became a uniform gel (the mass ratio of carbon material to PEDOT:PSS was 4:1 according to previous work [36]) for 1 h. The prepared PEDOT:PSS@3DFC-CNT paste was coated on the surface of the filter paper by a 4-sided film applicator (Zhuhai Kaivo Optoelectronic Technology Co., Ltd.) and naturally dried for about 12 h. As a control experiment, paste was also blade-coated on PET surface.

#### Fabrication of the All-Paper Pressure Sensors

The solution-processable all-paper sensor was assembled by stacking the top electrode on the top of the interdigitated electrode area and encapsulating them with adhesive tape. As a control experiment, Cu interdigitated electrode was also printed on PET substrates to assemble PET-based sensor.

### Characterization

Rheological properties of PEDOT:PSS@3DFC-CNT paste were measured using rheometer (Haake Mars 40). The morphology and microstructure of the prepared PEDOT:PSS@3DFC-CNT on filter paper and Cu-coated filter paper were characterized by scanning electron microscopy (SEM, SEISS Sigma 300). The 3D structure image of the PCCP was characterized by micro-computed tomography (MicroCT, Xradia 610 Versa). Deformation change of PCC during loading and unloading were characterized by field emission scanning electron microscope (FESEM, SEISS) with *in-situ* Micro Tensile Accessory (Deban). Optical images of the interdigitated electrodes were taken using an optical microscope (Olympus). The chemical compositions of the materials were studied using X-ray photoelectron spectroscopy (XPS, PHI 5000 Versa Probe equipped with a monochromatic Al Kα source). The adsorption–desorption isotherms of nitrogen (N_2_) (77 K) were measured by JWGB BK200C. For sensor characterization, current was measured using a Keithley 2400 SourceMeter at 0.01 V controlled by computer software. External pressure was applied using a homemade tension–compression apparatus, and a computer-controlled dynamometer (HP-5 and HP-50, China Handpi instruments). The resistances were measured by the two-point probe method with above-mentioned SourceMeter and the data were obtained using computer-controlled software. The sheet resistances were obtained by the four-point probe method measurement using HP504 (Guangzhou 4 Probes Tech.) with Keithley 2400 Sourcemeter. During the test, the film electrode is sampled at multiple points and the average values were taken to reduce the accidental error of the source meter measurement. For the sheet resistance (*R*_*sq*_) of the film, Eq. (1) was used to calculate:

1 $$R_{sq} = R \times F_{D/S} \times F_{W/S} \times F_{sp}$$

where *R* (Ω cm^−2^) is the measured resistive value, *D* (mm) is the sample diameter,* S* = 1.0 mm is the average probe spacing, *F*_*D/S*_ is the sample diameter correction factor and *F*_*W/S*_ is the sample thickness correction factor, *F*_*sp*_ = 0.1 is the probe spacing correction factor.

## Results and Discussion

### Design Principle and Structural Characterizations

**Scheme 1:**
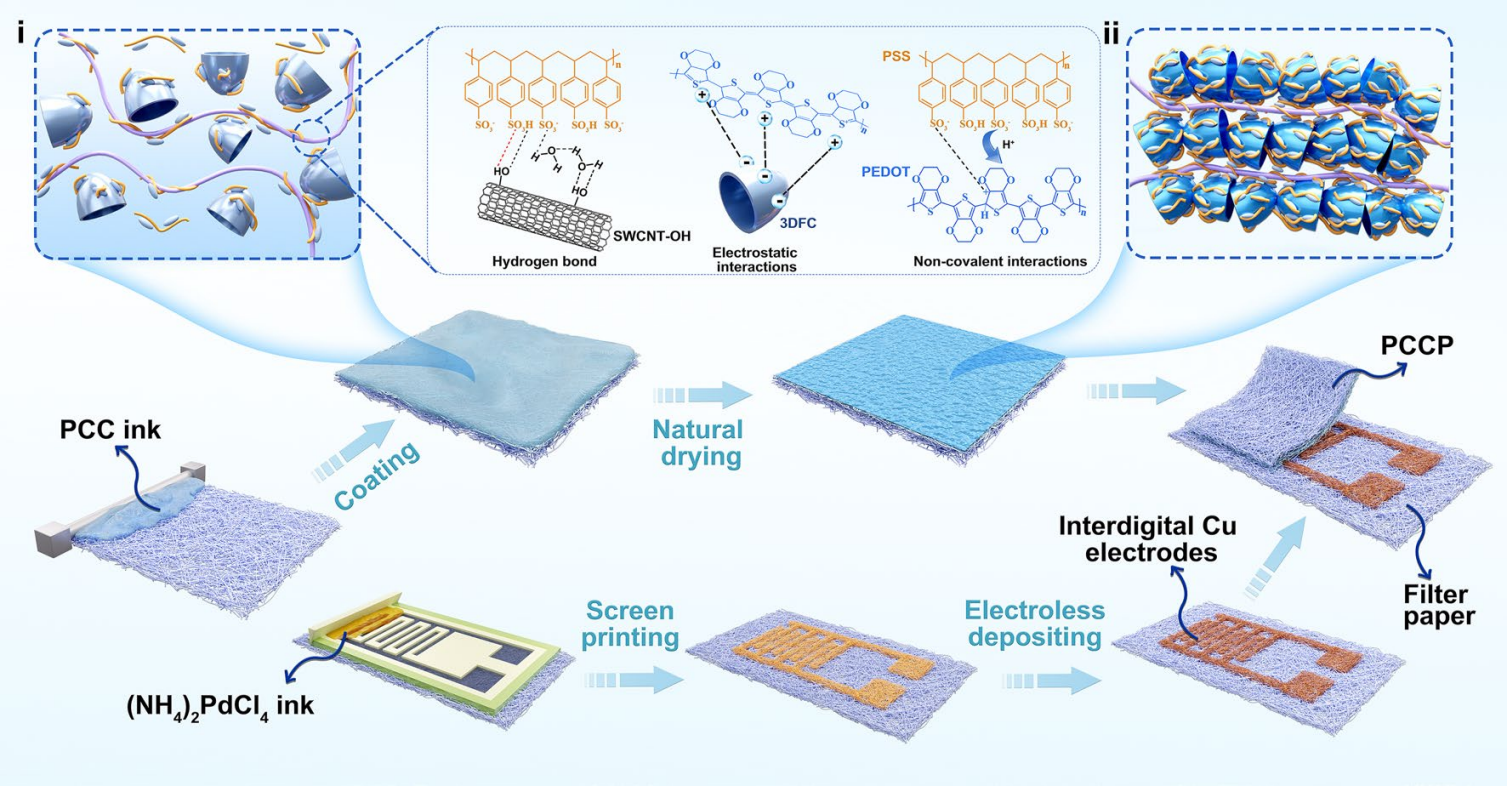
Schematic illustration of fabricating an all-paper pressure sensor by integrating a blade-coated top sensing electrode and a screen-printed bottom copper electrode. The inset (i) showing physical structural illustration of PEDOT:PSS@3DFC-CNT paste, and the enlarged view showing the corresponding interaction mechanism among material components; the inset (ii) showing physical structural illustration of dried PEDOT:PSS@3DFC-CNT as sensing material

**Fig. 1:**
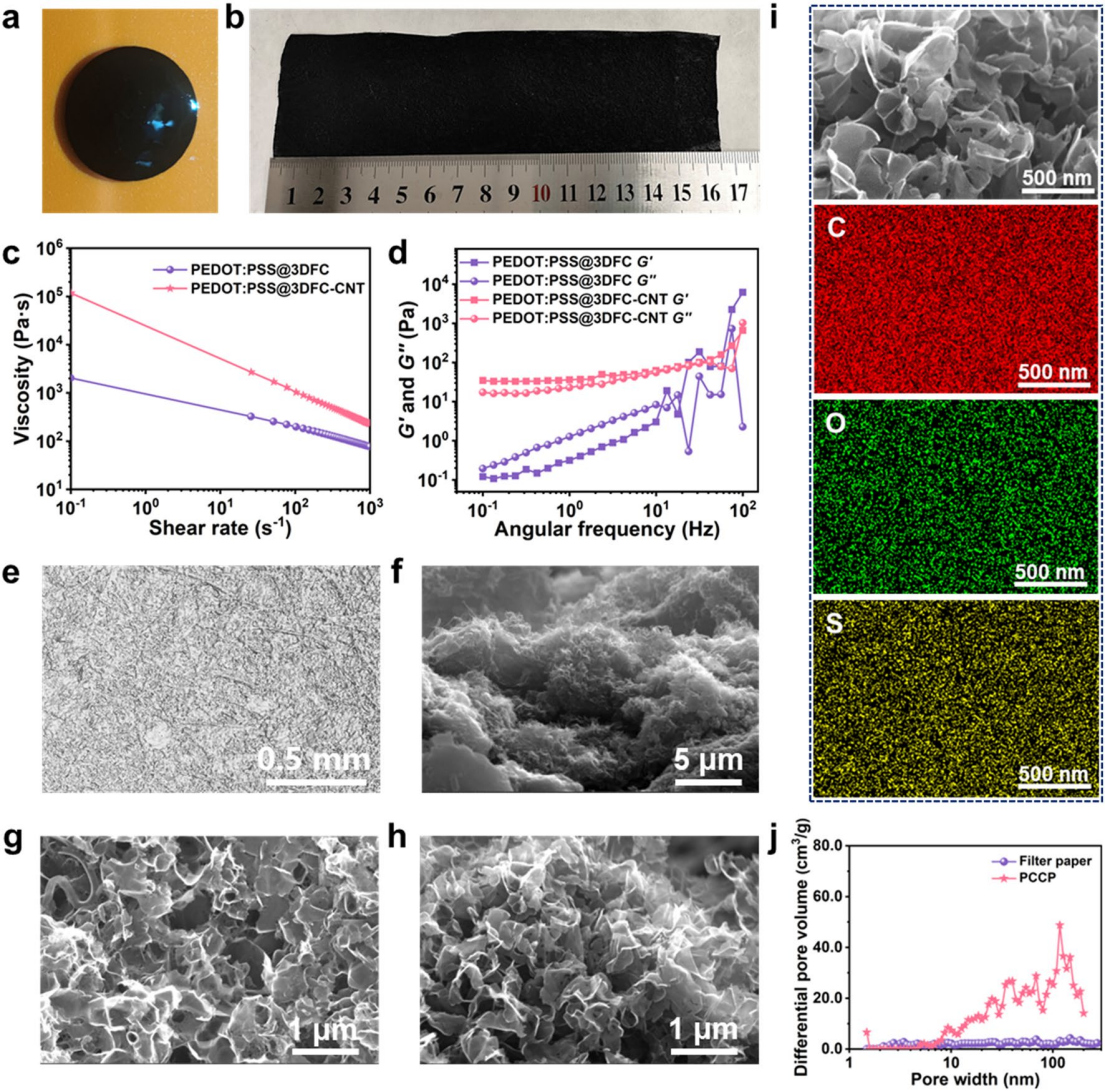
Characterization of blade-coated paste and PEDOT:PSS@3DFC-CNT sensing material. **a** Digital image of PEDOT:PSS@3DFC-CNT paste. **b** Digital image of PEDOT:PSS@3DFC-CNT on paper after blading coating and drying. **c** Changes in viscosities in pastes with/without CNTs as a function of shear rate, **d** changes of modulus recovery with angular frequency. **e** Top view of Micro-CT image of PCCP. **f** Tilted view SEM image of PEDOT:PSS@3DFC-CNT on paper. **g, h** Top-view and cross-sectional SEM images of PEDOT:PSS@3DFC-CNT. **i** Magnified image of panel h and the corresponding plan-scan SEM elemental distributions of C, O, and S. **j** Pore size distribution of filter paper and PCCP

The all-paper piezoresistive pressure sensor comprises a top sensing electrode and a bottom interdigital Cu electrode, as illustrated in Scheme 1. First, the sensing material paste of PEDOT:PSS-coated 3D carbon needs preparation for blade-coating. We selected three-dimensional frame carbon (3DFC) as the compressible framework, as depicted in the scanning electron microscope (SEM) image shown in Fig. S1, which consists of unique unit cells with a hollow shell structure. The conductive polymer PEDOT:PSS nanofibers dispersed in an aqueous solution are utilized to coat and bind the loose 3DFC by mixing them into the PEDOT:PSS dispersion (treated by 5.0 vol% dimethyl sulfoxide, DMSO). Simultaneously, CNTs, due to their special 1D tubular structure, are added into the dispersion and coated by PEDOT:PSS to enhance the cohesion and conductivity of the multiple composites. In this ternary composite paste, PEDOT:PSS acts as the binder and conductive agent, which is integrated through non-covalent interactions (such as electrostatic interactions and hydrogen bonds) between PEDOT and PSS polyelectrolytes [39, 40]. Due to the self-polarity of the PEDOT:PSS dispersion and the dimethyl sulfoxide treatment, positively charged PEDOT is more easily adsorbed on the negatively charged 3DFC surface through electrostatic interaction. PSS, containing sulfonic acid groups, tends to form hydrogen bonds with CNT-OH to encase the CNTs. As a result, a blade-coated paste with a gel state is formulated by mixing 3DFC, CNTs, and PEDOT:PSS in an aqueous solution, as shown in the digital image in Fig. 1a. Second, composite paste is blade-coated on filter paper due to the shear thinning effect. Interestingly, partial PEDOT:PSS can be squeezed into microfibers of paper during coating as the viscoelastic gel transforms into a fluid under shear. The shearing action potentially damages electrostatic interactions or hydrogen bonds between PEDOT(PSS) and 3DFC(CNT-OH), leading to a change in the paste state. After coating, the gel-state layer covers the surface and partially penetrates into the subsurface of the paper, and the physical and chemical structure recover, as illustrated in Scheme 1i. Through natural drying, a relatively dry composite forms because of the loss of water but retains cross-linked network structure (PEDOT:PSS@3DFC-CNT on paper named as PCCP; PEDOT:PSS@3DFC-CNT named as PCC), as illustrated in Scheme 1ii, which can be used as compressible sensing material. Figure 1b presents a piece of PCCP measuring 16.5 cm × 5 cm suggesting the potential for large-area fabrication, with the composite thickness above the paper around 180 μm, which is coated by a four-sided applicator with 750 μm gap clearance. Finally, the all-paper piezoresistive pressure sensor is constructed by integrating the PCCP top electrode and paper-based interdigited copper electrode. The bottom Cu electrode is created using our previously reported matrix-assisted catalytic printing method, involving the use of a screen-printing catalytic ink and electroless deposition of metal [38].

Physical and chemical characterization for paste and electrode material are conducted to verify the successful design for sensing material. The rheological properties (Fig. 1c) and shear modulus (Fig. 1d) of PEDOT:PSS@3DFC-CNT paste confirm our hypothesis. As a control experiment, paste without CNTs is also characterized. It is evident that CNTs are crucial for coating technology; they enhance the viscosity and elevate the loss modulus (*G′*), making it larger than the storage modulus (*G″*). The micro-computed tomography (Micro-CT) image demonstrates uniform dispersion of CNTs in the PEDOT:PSS@3DFC-CNT composite, connecting all the 3D skeletons as seen in Fig. 1e. Figure 1f presents a titled-view SEM image of PEDOT:PSS@3DFC-CNT, showing structural hills and valleys in dimension of several micrometers on the surface with nanostructures. Magnified top and cross-sectional views are displayed in Fig. 1g, h, where the unit cell resembles a half-eggshell derived from the salt template, with the feature size ranging from 200 to 500 nm and the wall thickness measuring several nanometers. These cells assemble to form numerous porous structures, retaining the original features of 3DFC after incorporation with PEDOT:PSS. Meanwhile, energy-dispersive spectroscopy (EDS) element mapping and elemental distribution of C, O, and S (Fig. 1i) reveal that PEDOT:PSS nanofibers uniformly coat on the surfaces of carbon materials. The chemical composition of PEDOT:PSS@3DFC-CNT is further confirmed by X-ray photoelectron spectroscopy (XPS) analysis in Fig. S2.

To demonstrate porosity and detect pore size, nitrogen adsorption–desorption isotherms (Fig. S3) and the corresponding pore size distribution (Fig. 1j) of filter paper and PCCP are analyzed. It is found that there is a significant increase in specific surface area from 1.4 m^2^ g^−1^ for filter paper to 15.6 m^2^ g^−1^ for PCCP. The porosity content increases across the entire detection range, with an average pore size of 140.4 nm, ranging from mesopores to macropores. These numerous pores provide compressible spaces for PEDOT:PSS@3DFC-CNT. To verify the compressibility of PEDOT:PSS@3DFC-CNT, we conducted three-point bending tests and *in situ* observed the sample’s compression and release under specific compressive force. The dynamic compression-release process can be seen in Movie S1 in the supporting information, where a 30.25 N force is applied. Figure S4 displays the initial state of the sample and its position after one compression-release cycle. The top positions of both states almost coincide, indicating the excellent compressibility and recovery capability of the PEDOT:PSS@3DFC-CNT composite. This suggests its potential as a sensing material for pressure sensors in terms of mechanics.

For bottom paper-based Cu electrode, there are 4 pairs of fingers (refer to Fig. S5) with an area of approximately 1.0 × 1.0 cm^2^. The width of each finger and the gap between the fingers are approximately 580 and 453 μm, respectively. The printed Cu film conformally coats on the cellulose fibers, maintaining the original fiber structure and roughness, as illustrated in Fig. S6. The measured finger resistance is less than 1.2 Ω cm^−1^ suggesting high conductivity. Similarly, for the top paper-based electrode, the abundant interstices and voids on a microscale between fibers create an effective capillary force, facilitating the uniform and secure adhesion of PEDOT:PSS@3DFC-CNT onto the cellulose fiber surface or between the fibers.

### Performance of All-Paper Pressure Sensors

**Fig. 2:**
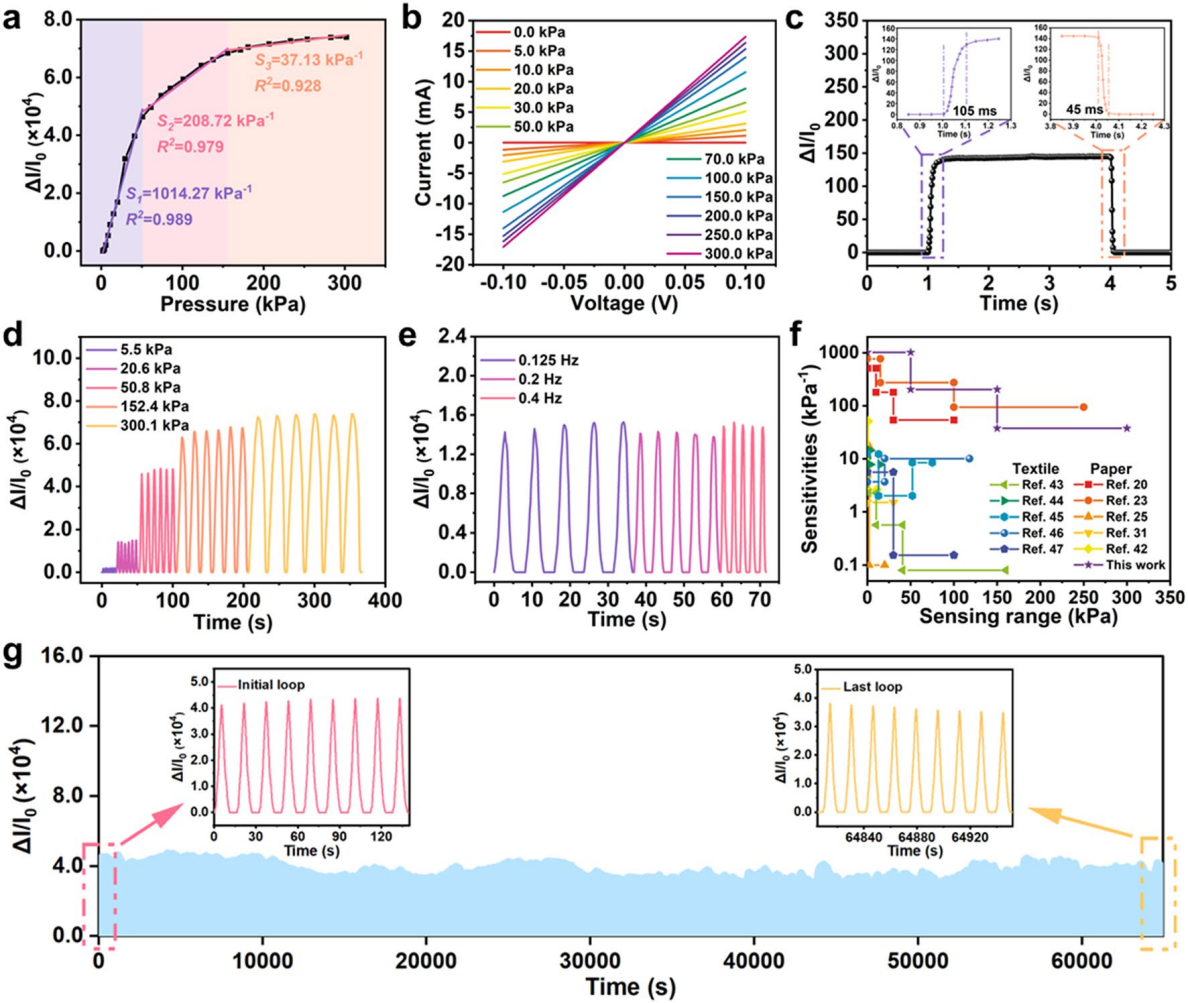
Sensing properties of the flexible all-paper pressure sensors. **a** Sensitivity of the all-paper piezoresistive pressure sensor. **b** *I*-*V* curves at different applied pressures. **c** Current response in a load-unload test at a pressure of 20 kPa, insets showing the responsive and recover time. **d** Sensing curves at different loading pressures. **e** Sensing curves at different loading frequencies. **f** Sensitivity and sensing range of all-paper pressure sensor compared to other paper-based and textile-based sensors reported in literatures (Refs. [19, 22, 24, 30, 41–46]). **g** Stability of the sensor during loading–unloading tests for 4000 cycles at a pressure of 50 kPa

Performance of all-paper pressure sensors are tested carefully after being constructed. The sensitivity curve displayed in Fig. 2a helps estimate the sensor's sensitivity, calculated using Eq. (2):

2 $$S = \frac{{\Delta I/I_{0} }}{\Delta P}$$

where *I*_*0*_ represents the sensor current without applied pressure, *ΔI* denotes the difference between the current after applying pressure and *I*_*0*_, and *ΔP* is the applied pressure. The curve's slope indicates the sensor sensitivity. Based on fitting results, the sensitivity can be subdivided into three parts: (1) for pressure within the range of 50 kPa, the sensitivity is 1014.27 kPa^−1^; (2) ranging from 50 to 150 kPa, it measures 208.72 kPa^−1^; (3) from 150 to 300 kPa, the sensitivity is 37.13 kPa^−1^. It is noteworthy that these sensitivities are optimized by adjusting the ratio of 3DFC to CNTs in ternary composite materials and the gap clearance of the four-sided applicator. The weight ratio of 3DFC to CNTs ranges from 1:1, 1:0.5, 1:0.33, 1:0.25, to 1:0, and the gap clearance varies from 250, 500, 750, to 1000 μm, respectively. A ratio of 1:1 leads to significantly higher paste viscosity due to a more CNTs content, making coating challenging. In contrast, a 1:0 ratio—lacking CNTs—not only impacts the coating process but also affects mechanical properties due to its fragility when under pressure. The samples with other different ratios are undergoing statistical analysis in terms of real coated thickness, sheet resistance, and the sensitivity of assembled sensors within the 0–50 kPa^−1^ range. Results indicate that the coating thickness exhibits minimal variation as the CNTs content decreases (Fig. S7a, Table S1), while the sheet resistance initially decreases then increases varying an order of magnitude (Fig. S7b, Table S2). Conversely, sensitivity shows an initial increase followed by a decrease as a whole (Fig. S7c, Table S3). Their corresponding surface morphologies can be found in the SEM images in Fig. S8. As the CNT content decreases, the morphology of carbon composite films shifts from the agglomeration state to a uniform state, and 3DFC becomes the primary component forming the main structure. When the mass ratio of 3DFC to CNTs is 1:0.33, its morphology lies between serious agglomeration and a skeleton-like 3DFC structure, where reasonable amount CNTs enhance the overall cohesion and conductivity of PEDOT:PSS@3DFC-CNT. Regarding different gap clearances adopted during blade coating, it is concluded that the actual coating thickness increases with an increasing of gap clearance, while the sheet resistance decreases; however, the sensitivity demonstrates an initial increase followed by a decrease. Consequently, the optimized sample is chosen with a ratio of 1:0.33 and a gap clearance of 750 μm, which is depicted in the sensitivity curve in Fig. 2a.

Figure 2b shows that the all-paper sensor maintains good linearity at 0, 5, 10, 20, 30, 50, 70, 100, 150, 200, 250, and 300 kPa, indicating that the sensor conforms to ohmic contact. The slope of the curve increases with increasing pressure, suggesting a decrease of resistance with increasing pressure. Response/recovery time is an important attribute of the sensor, and Fig. 2c shows that the response time of all-paper pressure sensor is 105 ms and the recovery time is 45 ms, suggesting its excellent responsive speed. As shown in Fig. 2d, different loading and unloading pressures are used in different sensitive sections to detect the current response for 6 times, e.g., 5.5, 20.6, 50.8, 152.4, and 300.1 kPa. The normalized currents (*ΔI/I*_*0*_) are positively correlated with the applied pressures, demonstrating good responsiveness and repeatability. In addition, the sensor maintains well repeatability at different frequencies, as shown in Fig. 2e. Three different frequencies of 0.125, 0.2, and 0.4 Hz are chosen to apply the loading–unloading cycles. The standard deviation of the current change over five randomly selected cycles is a maximum of 7.1%, which is within the error range. Then we cut the weighing paper to a weight of 0.001 N and fold it so that the contact surface is about 1 cm^2^. We slowly loaded it several times onto the top of sensor, and the sensor detects a change in current (Fig. S9), demonstrating a minimum detection limit of less than 10 Pa. Compared to other wearable pressure sensors based on porous rough paper [19, 22, 24, 30] and textile substrates [41–46], as-fabricated all-paper sensor takes on higher sensitivity and wider pressure response range (Fig. 2f). The detailed performance data can be found in Table S4. Its high performance should be due to the excellent conductivity of the active layer and the special coupling structure of the PEDOT@3DFC-CNT. Detailed analysis will be discussed in the next section. Finally, the cyclic stability of the sensor is also tested. During 4000 loading–unloading cycles at a pressure of 50 kPa (Fig. 2g), the current response remains consistent, and the initial nine cycles and the last nine cycles maintain similar normalized current profiles. The peak current drops by only about 15.1%, which demonstrated the reliable cyclic stability of our fabricated sensor. After being shelved for 150 days, all-paper sensor is tested and shows similar sensitive property with that in Fig. 2a (Fig. S10), demonstrating the long-term stability of the as- fabricated all-paper piezoresistive pressure sensor.

### Sensing Mechanism of All-Paper Piezoresistive Pressure Sensors

To elucidate the sensing mechanism of our fabricated all-paper piezoresistive pressure sensor, cross-sectional SEM images of sensor (Fig. S11a) and top PCCP electrode (Fig. S11b, c) are captured. The protrusion microstructures on the PCCP surface are clearly visible (Fig. S11d–f). The formation reason could be losing H_2_O, structural shrinkage and partial domain sinking derived from porous and rough structure of paper, during natural drying. The micro/nano hierarchical structure lays the foundation for the large interfacial deformation of the active layer. Then, we analyze a cross-sectional SEM image and the corresponding EDS element mapping image and Cu, C, O, and S distribution of the sensor, as shown in Fig. S11g, h. In Fig. S11g, the bottom electrode with Cu on the filter paper and the top sensing layer of PCCP can be clearly seen. Because the cross-section is formed by cutting the sensor along with the Cu finger, a thin continuous layer of Cu element can be seen. For top electrode, elemental concentrations of C and O display the obvious line of demarcation, carbon concentration below white dot line is more than that above white dot line, while oxygen content is exactly the opposite. It is attribute to the high carbon content of 3DFC and CNTs in PEDOT:PSS@3DFC-CNT and low oxygen content as compared with cellulose paper. Based on the distribution of C and O, it is deduced that the white dot line in Fig. S11g, h is the interface of PEDOT:PSS@3DFC-CNT and filter paper. At the same time, PEDOT:PSS also partially penetrate into filter paper and coat onto microfibers which is derived from the distribution of elemental S in filter paper. This can be traced back to the blade-coating process of paste.

In the previous section, PEDOT:PSS@3DFC-CNT has been proved having the compressibility via the *in situ* three-point bending test. To further verify the impacts of PEDOT:PSS@3DFC-CNT and filter paper on sensitivity, respectively, flexible PET is used as top and bottom substrates to make PET-based sensor for comparison. The sensitivity of the sensor within 0–50 kPa is tested, and the results are shown in Fig. S12a. The sensitivity of all-paper sensor (1014.27 kPa^−1^) is remarkedly better than that of the all-PET sensor (47.34 kPa^−1^). This phenomenon can illustrate from two points: (1) pure ternary composite of PEDOT:PSS@3DFC-CNT can indeed act as sensing material for pressure sensor; (2) paper with microfibers and porosity play an important role for significantly improving sensitivity. As shown in Fig. S6, unlike the smooth and flat surface of PET, the paper fibers have a cylindrical structure, which can provide larger gaps between fibers leading to a perfect deformation space for the sensing layer and anchored enhancement for PEDOT:PSS@3DFC-CNT on paper. Here, PEDOT:PSS coat on the microfibers where is near the interface between PCC and paper. To detect the sensing performance of PEDOT:PSS coated microfibers, similar controlled experiment is conducted by constructing all-paper sensor with top electrode of PEDOT:PSS coated filter paper. In Fig. S12b, we compare the sensitivities with detecting range from 0 to 300 kPa between PCCP and PEDOT:PSS coated paper. For the later sensor, the sensitivity can be divided into two sections at the point of 100 kPa. Before 100 kPa, the sensitivity is 4.37 kPa^−1^, which is attributed to the contact of rough Cu electrode and PEDOT:PSS coated paper; after 100 kPa, the sensitivity increases to 34.16 kPa^−1^, which can be attributed to the compress of Cu and PEDOT:PSS coated microfibers. As comparison, it is found that PCC based all-paper sensor has the similar sensitivity (37.13 kPa^−1^) with PEDOT:PSS based all-paper sensor after 150 kPa. Based on this, we judge that conductive microfibers of paper contribute the terminal sensitivity of all-paper sensors. According to the aforementioned experiments and analyses, we conclude that PCC on porous paper makes outstanding contribution for sensitivity in the range of 0–150 kPa. The coupling effect between PCC and porous paper results in the very rough surface with micro/nano hierarchical structures (Figs. 1f and S11a–f), further increasing the sensitivity within the range of 50 kPa, while the all-PET sensing layer has smaller rough surface because the flatten surface of PET cannot absorb water and PEDOT:PSS. In the pressure range between 50 and 150 kPa, *S*_*2*_ = 208.72 kPa^−1^, the high sensitivity should be derived from the internal porous structure of PEDOT:PSS@3DFC-CNT (Fig. 1h, j).

**Fig. 3:**
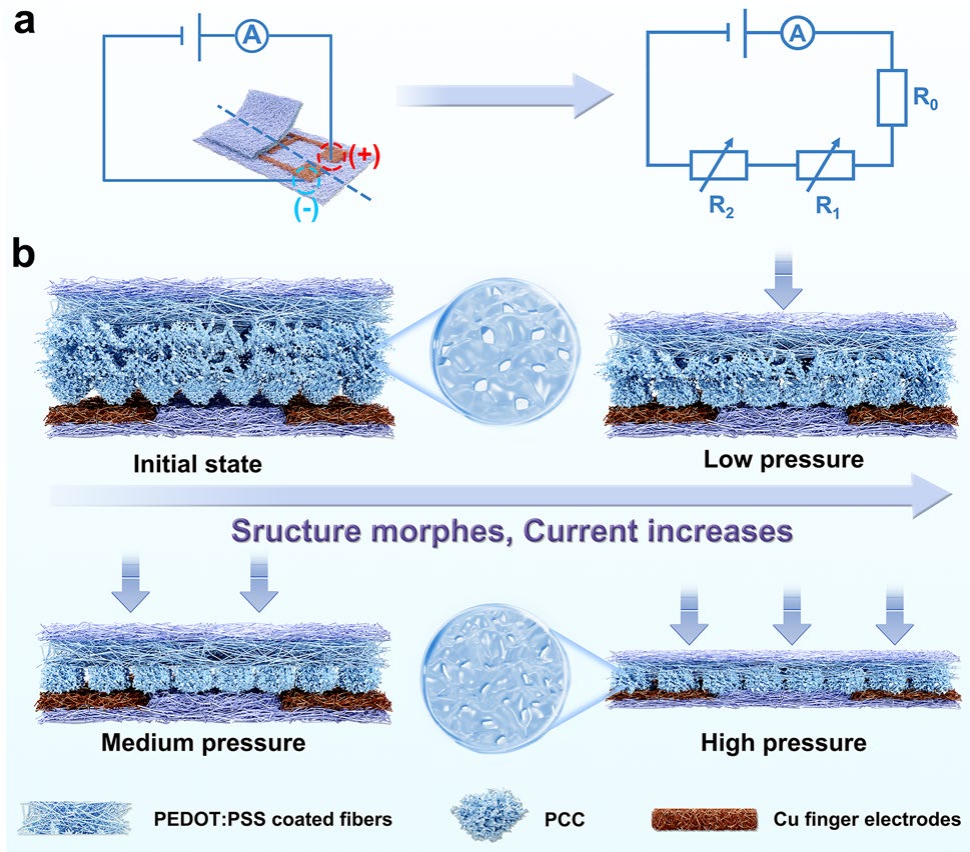
Working mechanism of all-paper piezoresistive pressure sensors. **a** Equivalent circuit diagram of the all-paper sensor. **b** Schematic illustration of operating mechanism for all-paper resistive pressure sensor during loading process

Based on above-mentioned analysis, the sensing mechanism of our fabricated all-paper sensors is explained as follows. The equivalent circuit diagram and schematic illustration are shown in Fig. 3a, b. The whole sensor can be equated as a series circuit, and the total resistance (*R*) is mainly provided by intrinsic resistance of the connection wire and copper electrode (*R*_*0*_), the contact resistance (*R*_*1*_) between the PCCP and the copper interdigital electrode, and resistance of overall structure in PCCP (*R*_*2*_), in which *R*_*0*_ is relatively fixed, *R*_*1*_ and *R*_*2*_ are variable with deformation. Due to the sub-millimeter width and the micro-scale rough surface of the bottom Cu electrode and hierarchical micro/nano-structure surface with hills and valleys of top PCCP electrode, there are fewer interfacial contact points between them at the initial state. Therefore, the initial contact resistance *R*_*1*_ is extremely high, making the initial current (*I*_*0*_) lowest. When low pressure is loaded, the sensing layer and the electrode fingers start to quickly contact, the amount of current change (*ΔI*) increasing significantly until the interface is in full contact, thus providing high sensitivity (*S*_*1*_ in Fig. 2a). As the pressure is further increased, the internal nanoporous structures of PCC are gradually compressed, PEDOT:PSS coated 3DFC deform and contact each other tightly, leading to more conductive paths and a reduction in *R*_*2*_. During this period, the sensitivity (*S*_*2*_ in Fig. 2a) is lower than in the first stage. As pressure continues to be applied during the final pressurization stage, the contact interface between the top and bottom electrodes and PCC become compressed to a saturated state. At this juncture, the change in contact of the conductive microfibers within the paper becomes the primary factor contributing to the decrease in *R*_*2*_ under high pressure. The sensitivity (*S*_*3*_ in Fig. 2a) also reaches a certain threshold. Consequently, the sensitivity of the all-paper sensor can be segmented into three regions ranging from 0 to 300 kPa, as depicted in Fig. 2a, with each deformation stage significantly extending the operational range. It is essential to note that PCCP not only enhances sensitivity due to its multiscale hierarchical rough surface but also broadens the pressure range from 50 to 300 kPa owing to its internal nanoscale porous structures and conductive microfibers. By integrating the bottom electrode, the multiscale hierarchical interface coupled with nanoporous structures results in ultrahigh sensitivity and a wide detection range for the all-paper pressure sensor. Notably, "multi-scale" encompasses nano-, micro-, and submilli-scale dimensions.

### Human Physiological Monitoring

**Fig. 4:**
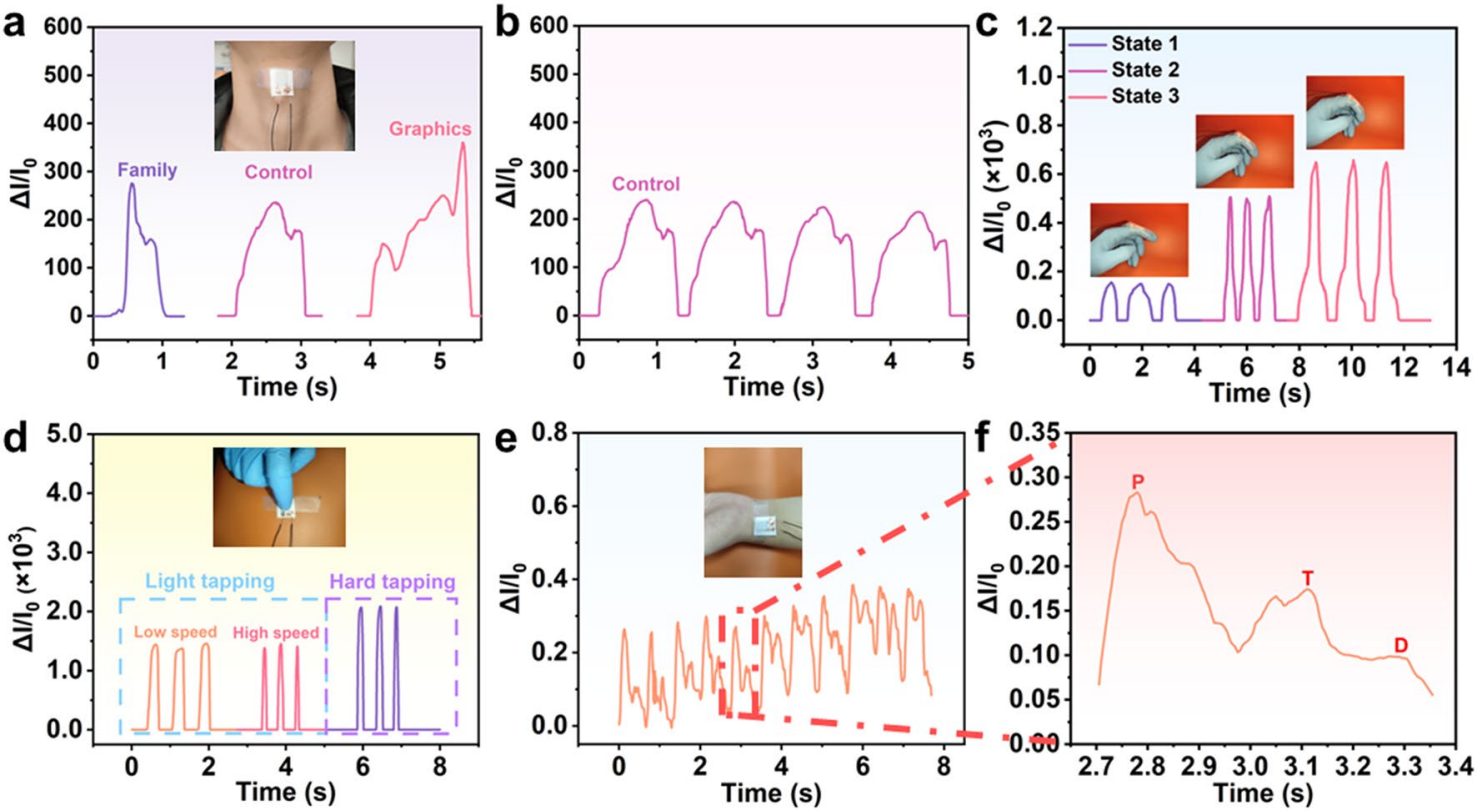
Application demonstration of all-paper pressure sensors. **a** Sensor attached to the laryngeal node and the vocal cord vibrates during articulation (Family, Control, Graphics), thus causing a corresponding current change. **b** View of the current change during the reading of the same word (Control). **c** Current response caused by bending the index finger with a fast motion and increasing bending angle when the sensor is loaded on the finger. **d** Change in the current signal of the sensor on a table when the finger tapping with different force and frequencies. **e** Sensor bound to the wrist to detect pulse-induced vibration, and **f** magnified view of a peak taken from Fig. 4e

For human physiological signals, the pressure exerted by gently manipulated objects typically falls between 1 and 10 kPa, while the pressure from pulses, body weight, and joint movements ranges from 10 Pa to 300 kPa. Clearly, flexible all-paper pressure sensors are fabricated to enable effective monitoring of human physiological signals. In the postoperative recovery of vocal cord-related diseases, detecting voice pronunciation is essential for returning to normal life after surgery. Monitoring postoperative recovery by assessing different word pronunciations is crucial [47]. Here, the all-paper sensor is fixed to the glottal node to detect human vocal fold vibrations, as shown in the inset of Fig. 4a. We sequentially pronounce "FAMILY" and "CONTROL" and "GRAPHICS", converting the mechanical signals of the glottal knot vibration into electrical signals. The shape of the curve clearly shows the difference in electrical signals displayed by different words, demonstrating the sensor's excellent ability to distinguish different words. In Fig. 4b, the same word "CONTROL" is read repeatedly, and the results shows that the electrical signals of the sensors for the same word are similar, reflecting the excellent stability of the sensor for pronunciation recognition. Moreover, the large movements of the patient's joints and fingers also affect the diagnosis, treatment, and evaluation of the patient's condition [48]. Our all-paper sensor is affixed to the knuckle and bent to various angles of approximately 15°, 60°, and 75° by bending the index finger. Figure 4c illustrates that the current signal increases proportionally with the bending angle, indicating the sensor's excellent flexibility and stability. In disease diagnosis and treatment, the movement of fingers is also an important evaluation criterion [49]. In Fig. 4d, a touch test of the sensor is conducted, with slow and fast tapping using varying force. Additionally, different tapping forces are also tested to fully evaluate the tapping state of fingers on the sensor surface. The results demonstrate the sensor's stability and responsiveness, whether subjected to different tapping speeds or tapping forces, effectively capturing signal of finger movement, providing an effective evaluation method for disease diagnosis and treatment. To explore the sensor's application in detecting tiny forces, we employed it for wrist pulse monitoring, as depicted in Fig. 4e. Wrist pulse signals are indicative of cardiovascular health and can reflect the patient's health status during physical activity [50]. The sensor detected 11 pulse beats in 10 s (66 beats per minute), showcasing its repeatability in pulse signal detection. In Fig. 4f, three distinct rhythm patterns—percussion (*P*), tidal (*T*), and diastolic (*D*)—are evident in a single pulse wave, highlighting typical pulse wave characteristics. These demonstrations underscore the excellent performance of our flexible all-paper sensors in monitoring the human body across a range of pressures, from low to high, with rapid response times. This capability holds great promise for wearable and flexible electronic products.

### Demonstration of Large-Area All-Paper Sensor Array

**Fig. 5:**
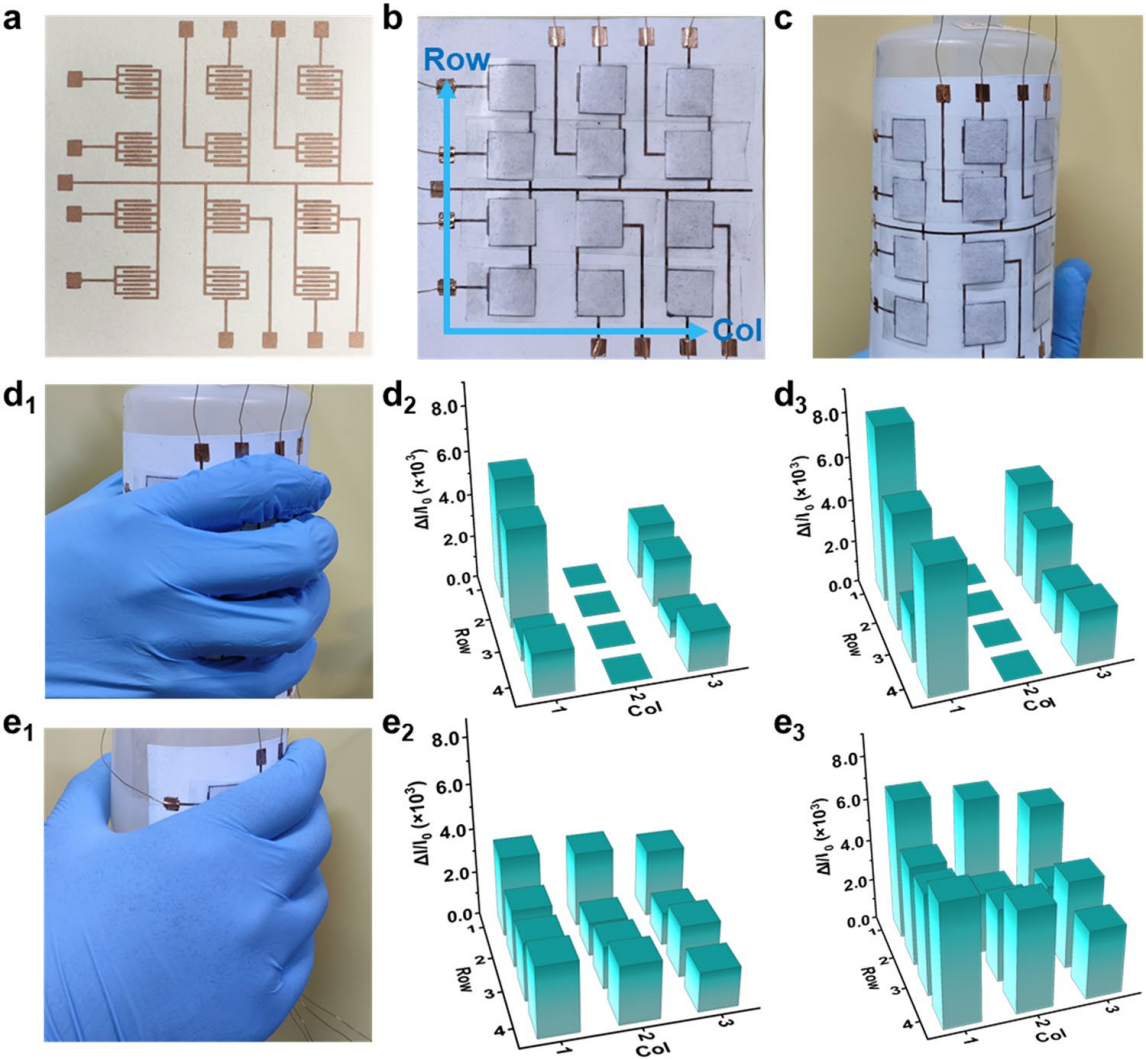
Demonstration of the all-paper sensor array used to resolve spatial pressure. **a** Digital image of printed Cu circuit with electrodes array, **b** digital image of all-paper sensor array with 3 × 4 single sensors integrated by the Cu circuit, and **c** digital image of flexible sensor array attached on wash bottle. **d**_**1**_ Digital image of holding a bottle with sensor array in hand by a hollow-hand gesture, and the corresponding 3D histograms of current response for **d**_**2**_ empty bottle and **d**_**3**_ filled water bottle, respectively. **e**_**1**_ Digital image of holding a bottle with sensor array in hand by a full-hand gesture, and the corresponding 3D histograms of current response for **e**_**2**_ empty bottle and **e**_**3**_ filled water bottle

Postoperative recovery for patients is a crucial aspect of medical care, especially for individuals recovering from conditions like Parkinson’s disease. Usually, patients with Parkinson’s disease have difficulty moving their finger joints during postoperative recovery. For the same patient, the spatial distribution of grip force will change during the recovery process [51]. By analyzing the differences in spatial force distribution, basic predictions can be made for disease diagnosis and treatment [52]. By monitoring the patient's hand grasping posture, it becomes possible to further evaluate their physical recovery progress. To demonstrate the applicability of the developed all-paper pressure sensor in detecting human body signals related to spatial mechanics, a recyclable flexible sensing pad is constructed [53, 54]. Using screen printing technology, an integrated Cu electrode array is printed on a large-area paper substrate. Figure 5a displays a 3 × 4 electrode array on a single piece of paper. Each electrode in Fig. 5b is covered by a PCCP layer measuring 1 × 1 cm^2^, creating an integrated flexible sensing pad (10 × 10 cm^2^). The resistance change in each sensing unit provides real-time feedback on the current signal to detect 3D force. In Fig. 5c, the sensing pad is attached to a plastic washing bottle, demonstrating its exceptional flexibility. To illustrate the sensor array's response to different human hand gestures, various hand gestures are used to grasp an empty bottle, revealing the spatial force distribution. Figure 5 d_1_ depicts the use of a hollow hand gesture, while Fig. 5e_1_ shows a full-hand grasping gesture. The corresponding electrical signal changes can be observed in Fig. 5d_2_, e_2_, respectively, reflecting the distribution of grasping force. The current change is zero in column 2 of Fig. 5d_2_, indicating the hollow hand gesture, while the current change is similar among the three columns in Fig. 5e_2_, suggesting the full-hand gesture. The related 3D histograms demonstrate the flexible sensing pad’s excellent ability to distinguish gripping postures. Furthermore, the gripping force distribution of the hand is simultaneously distinguished when the bottle is filled with water. Figure 5d_3_, e_3_, respectively, presents their current change. Compared with an empty bottle, the current intensity of every sensor notably increases, indicating an enhancement of gripping force overall. Moreover, to demonstrate the normal grasping and abnormal grasping conditions during postoperative recovery, the grasping state of patients with Parkinson’s disease has been simulated (Movie S2) and the corresponding current curves of the sensor at different jitter locations has also been collected (Fig. S13a, b). In addition, different jitter amplitudes and frequencies (Fig. S13c, d) under grasping posture are also recorded to further explore the impact of different hand jitter on the current signal of the sensing array when grasping. In summary, the 3D histograms illustrate that the flexible large-areal sensing pad effectively provides feedback on the spatial distribution and intensity of force, which provides a reference for providing some guiding data for recovery after Parkinson's surgery.

## Conclusions

In summary, our research pioneers a novel fabrication strategy for an all-paper pressure sensor, setting a benchmark in sensitivity and detection range. Capitalizing on the natural roughness and porosity of filter paper, our design integrates PEDOT:PSS@3DFC-CNT to create a structural coupling with surface micro/nano structures and porous nanostructures. This is complemented by a copper finger electrode, enhancing the sensor's multi-scale hierarchical interface, pivotal for its outstanding performance. The heart of the sensor's superior functionality lies in its unique multi-scale hierarchical deformation mechanism. This is facilitated by a robust 3D hollow carbon framework, providing resilience, while PEDOT:PSS and CNTs contribute to conductivity and structural strength. The sensor's remarkable sensitivity of 1014 kPa^−1^, wide response range up to 300 kPa, and low detection threshold below 10 Pa, significantly outperform most existing paper-based counterparts. Apart from its technical excellence, the sensor demonstrates rapid response, reliable repeatability across diverse pressure intensities and frequencies, and maintains robustness for 4000 cycles. Its proficiency in monitoring intricate physiological signals positions it as a prime candidate for next-generation wearable health monitors, robotics, and Internet of Things applications. The sensor's low-power requirement and compatibility with scalable, low-cost production processes further amplify its practical appeal. Moreover, the integration of solution processing ensures compatibility with printed electronics, heralding a new era for smart, sustainable electronics. This research not only pushes the boundaries of sensor technology but also lays the groundwork for future innovations in wearable and green electronics.

## Supplementary Information

Below is the link to the electronic supplementary material.

Supplementary file1 (MP4 10042 KB)

Supplementary file2 (MP4 23735 KB)

Supplementary file3 (DOCX 9686 KB)
