# Supplementary material for: Blade-Coated Porous 3D Carbon Composite Electrodes Coupled with Multiscale Interfaces for Highly Sensitive All-Paper Pressure Sensors
Source: Nanomicro Lett. 2024 Aug 13;16:267. doi: 10.1007/s40820-024-01488-0 (PMC11319548; doi:10.1007/s40820-024-01488-0)
Supplement: Supplementary file 3 — Supplementary file3 (DOCX 9686 KB) [file 40820_2024_1488_MOESM3_ESM.docx]

**Blade-Coated Porous 3D Carbon Composite Electrodes Coupled with Multiscale Interfaces for Highly Sensitive All-Paper Pressure Sensors**

Bowen Zheng^1^, Ruisheng Guo^1,^*, Xiaoqiang Dou^1^, Yueqing Fu^1^, Bingjun Yang^2,^*, Xuqing Liu^1,4,^* and Feng Zhou^3,4^

^1^ State Key Laboratory of Solidification Processing, Center of Advanced Lubrication and Seal Materials, School of Materials Science and Engineering, Northwestern Polytechnical University, Xi’an, Shanxi 710072, China

^2^ Research Center of Resource Chemistry and Energy Materials, State Key Laboratory of Solid Lubrication, Lanzhou Institute of Chemical Physics, Chinese of Academy of Sciences, Lanzhou 730000, China

^3^ State Key Laboratory of Solid Lubrication, Lanzhou Institute of Chemical Physics, Chinese Academy of Sciences, Lanzhou 730000, China

^4^ Shandong Laboratory of Advanced Materials and Green Manufacturing at Yantai, Yantai, Shandong 264006, China

*Corresponding author. E-mail: [guoruisheng@nwpu.edu.cn](mailto:guoruisheng@nwpu.edu.cn); [yangbj@licp.cas.cn](mailto:yangbj@licp.cas.cn); [xqliu@nwpu.edu.cn](mailto:xqliu@nwpu.edu.cn);

**Figure S1**.SEM image of pure 3DFC.


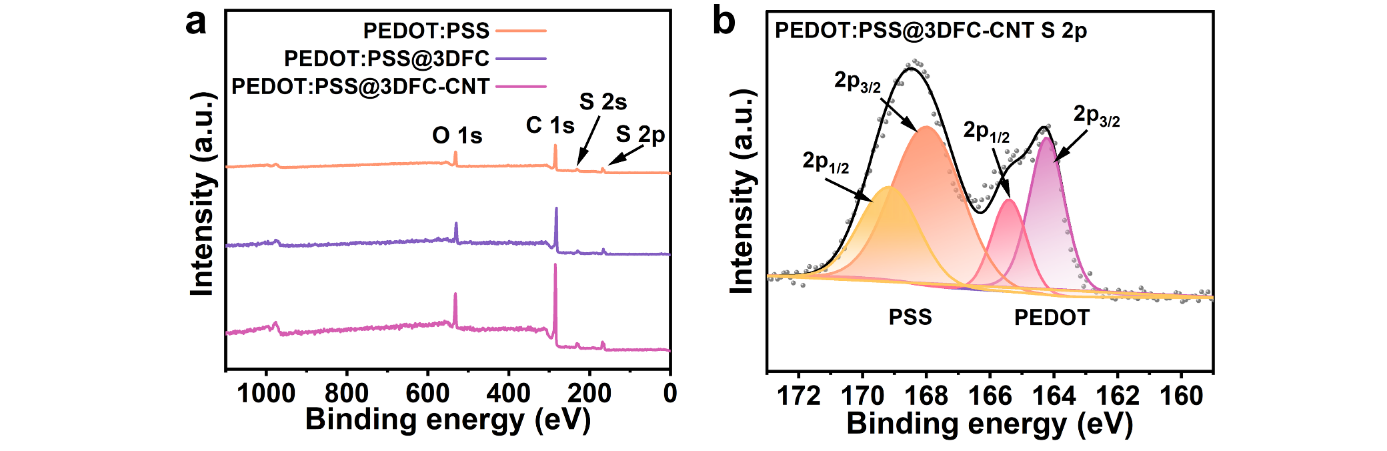


**Figure S2.** (a) Full XPS spectra of PEDOT:PSS@3DFC-CNT with different ratio of 3DFC to CNTs. (b) Fine XPS spectrum of S 2p in PCCP.

**Figure S2a** shows the XPS spectrum of the thin film. After adding 3DFC to PEDOT:PSS, the energy spectrum of PEDOT:PSS@3DFC showed the unique S spectrum line of PEDOT:PSS, proving the successful incorporation of PEDOT:PSS. At the same time, the C/S ratio in PEDOT:PSS@3DFC is smaller than the PEDOT:PSS standard of 6.0, which may be attributed to the π-π* stacking interaction between the PEDOT main chain and the 3DFC surface. When we continue to add CNT to PEDOT:PSS@3DFC, the proportion of O in the energy spectrum greatly increases. This is due to the large amount of hydroxyl groups in SWCNT-OH, which further illustrates the successful addition of CNT. **Figure S2a** shows fine XPS spectrum of S 2p in PCCP. Four peak positions can be observed in 169.1eV, 167.9eV and 165.4eV, 164.2eV, which correspond to S 2p_1/2_ and S 2p_3/2_ in PSS and S 2p_1/2_ and S 2p_3/2_ in PEDOT respectively.

**Figure S3.** Nitrogen adsorption-desorption isotherms curves of filter paper and PCCP, respectively.


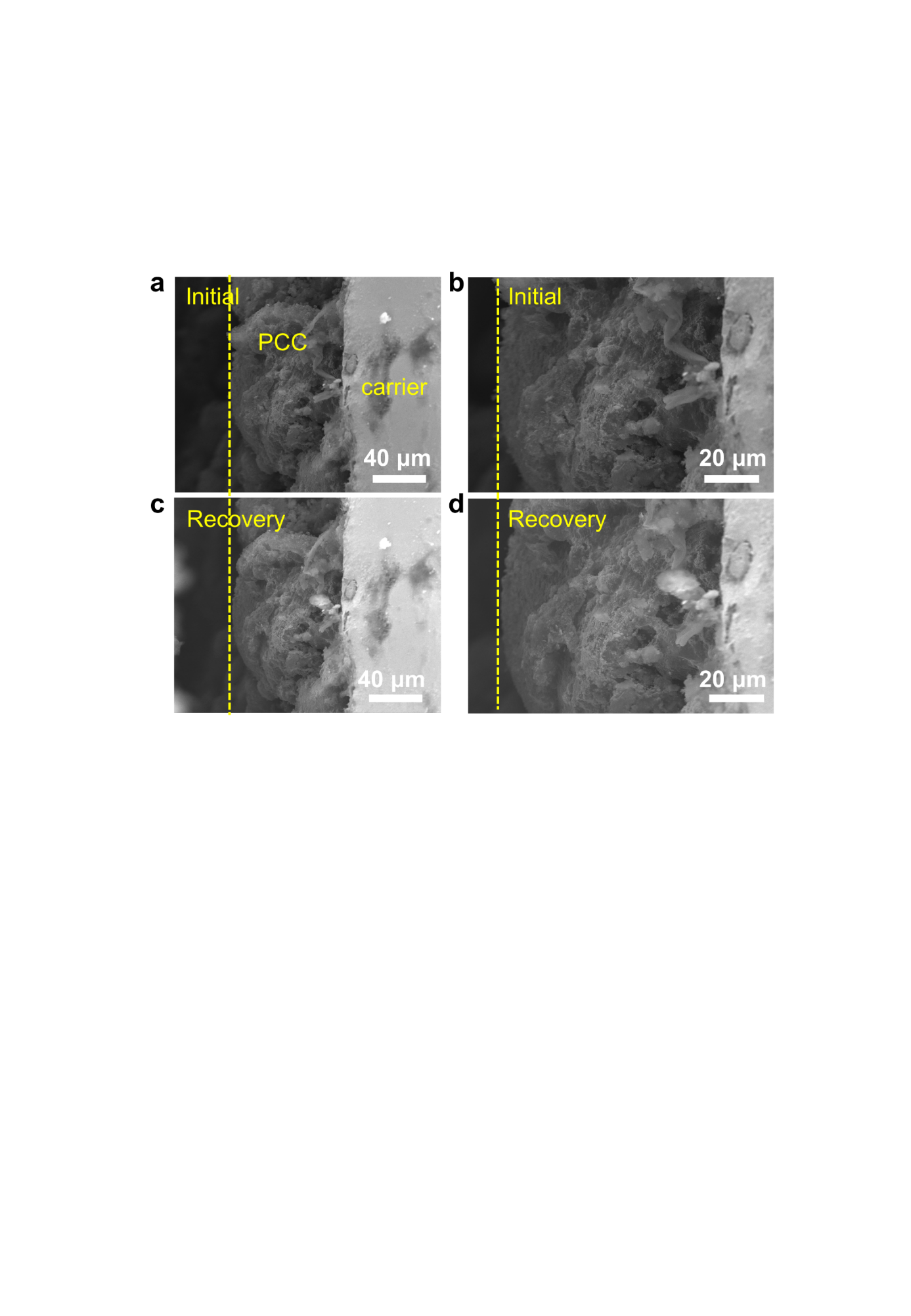


**Figure S4.** **FESEM image of the PCC thin film electrodes.** (a) Initial state of PCC. (b) Enlarge image of Figure a. (c) Recovery state after loading 30.25N. (d) Enlarge image of Figure c.


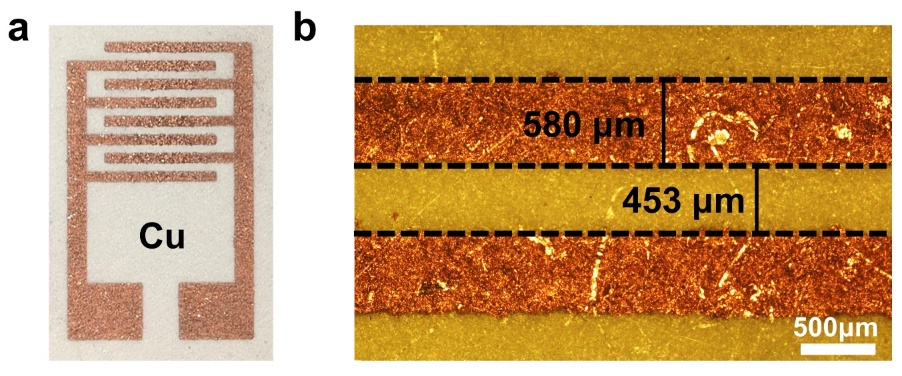


**Figure S5.** (a) Digital image and (b) optical image of interdigital Cu electrode printed on filter paper.


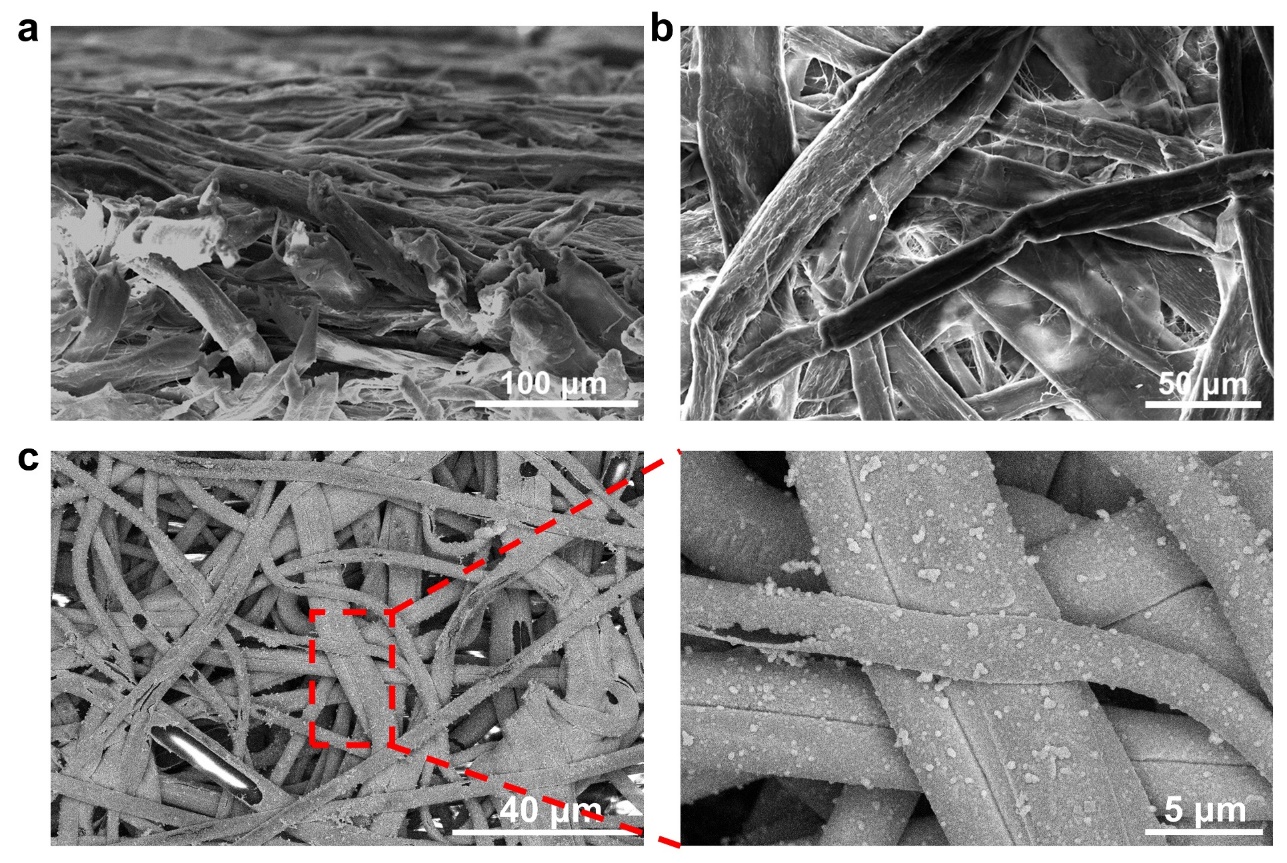


**Figure S6**. (a) SEM image and (b) cross-section SEM image of filter paper.

(c) SEM image of the filter paper with Cu cross fingers.


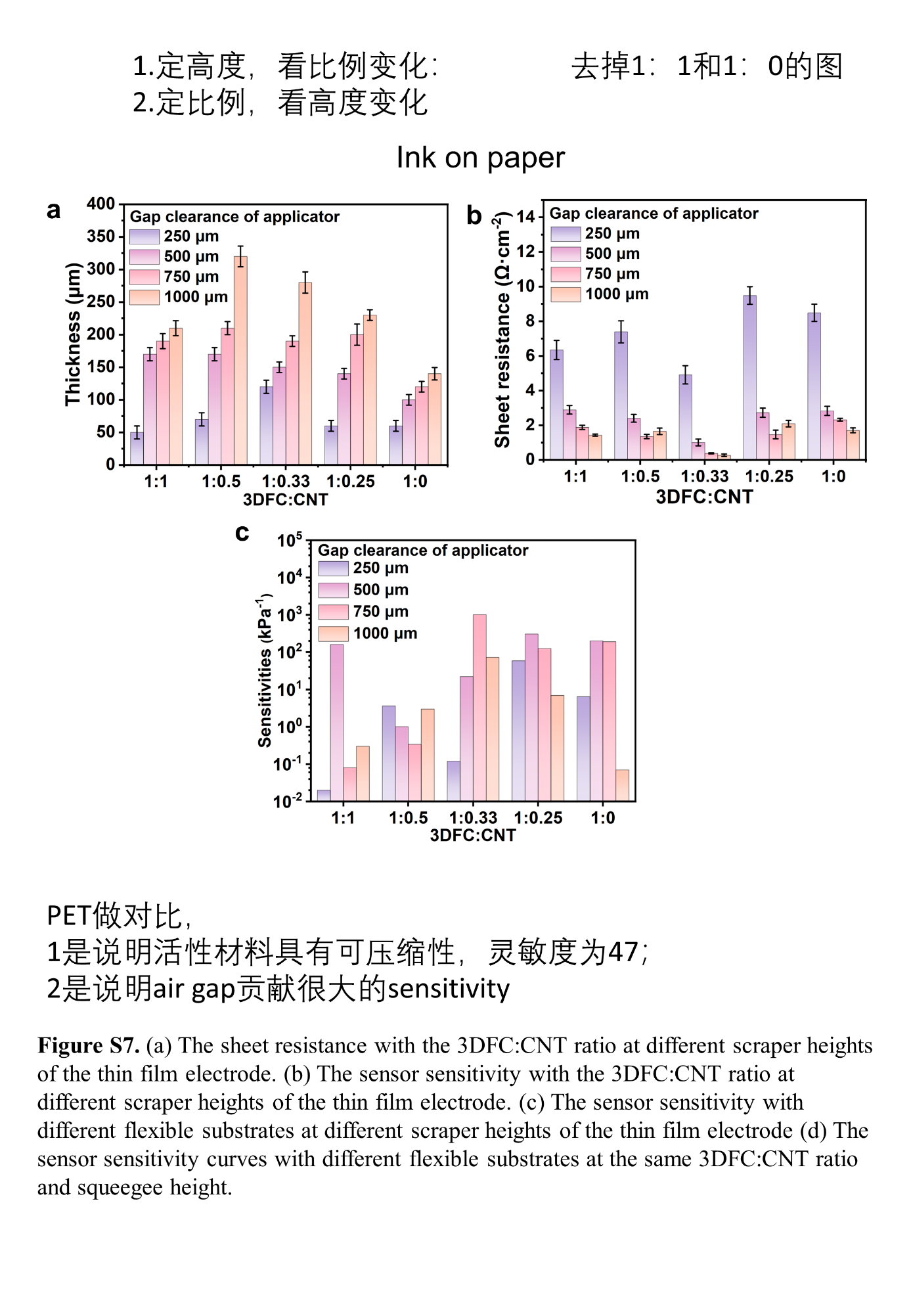


**Figure S7.** (a) Thickness of PEODT@3DFC-CNT film with varying 3DFC:CNT ratios at different gap clearances. (b) Sheet resistance of PEODT@3DFC-CNT film with varying 3DFC:CNT ratios at different gap clearances. (c) Sensor sensitivity of PEODT@3DFC-CNT film with varying 3DFC:CNT ratios at different gap clearances.

**Table S1.** Thickness of PEDOT@3DFC-CNT films (μm).

| Gap clearance  3DFC:CNT | 250 μm | Er± | 500 μm | Er± | 750 μm | Er± | 1000 μm | Er± |
| --- | --- | --- | --- | --- | --- | --- | --- | --- |
| 1:1 | 50 | 10.0 | 170 | 10.0 | 190 | 11.5 | 210 | 11.5 |
| 1:0.5 | 70 | 10.0 | 170 | 10.0 | 210 | 10.0 | 320 | 15.8 |
| 1:0.33 | 120 | 10.0 | 150 | 8.2 | 190 | 8.2 | 280 | 16.3 |
| 1:0.25 | 60 | 8.2 | 140 | 8.2 | 200 | 16.3 | 230 | 8.2 |
| 1:0 | 60 | 8.2 | 100 | 8.2 | 120 | 8.2 | 140 | 9.6 |

**Table S2**. Sheet resistance of PEDOT@3DFC-CNT films (Ω·cm^-2^).

| Gap clearance  3DFC:CNT | 250 μm | Er± | 500 μm | Er± | 750 μm | Er± | 1000 μm | Er± |
| --- | --- | --- | --- | --- | --- | --- | --- | --- |
| 1:1 | 6.345 | 0.552 | 2.891 | 0.244 | 1.878 | 0.121 | 1.431 | 0.060 |
| 1:0.5 | 7.392 | 0.635 | 2.405 | 0.226 | 1.352 | 0.126 | 1.654 | 0.186 |
| 1:0.33 | 4.907 | 0.527 | 1.006 | 0.197 | 0.377 | 0.033 | 0.272 | 0.069 |
| 1:0.25 | 9.486 | 0.510 | 2.727 | 0.268 | 1.469 | 0.247 | 2.096 | 0.186 |
| 1:0 | 8.490 | 0.496 | 2.828 | 0.263 | 2.315 | 0.080 | 1.705 | 0.141 |


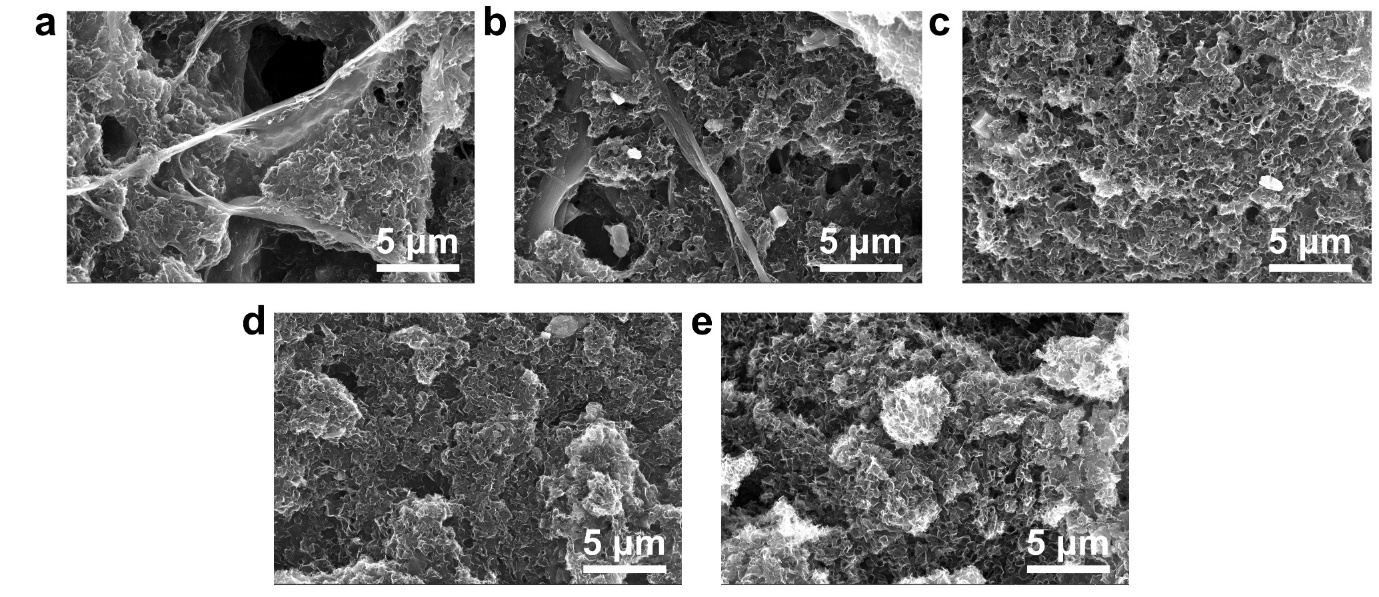


**Figure S8.** SEM images of PEDOT:PSS@3DFC-CNT containing 3DFC and CNTs with ratio of (a) 1:1, (b) 1:0.5, (c) 1:0.33, (d) 1:0.25, and (e) 1:0, respectively.

**Table S3**. Sensitivity of all-paper sensors within 0-50 kPa (kPa^-1^).

| Gap clearance  3DFC:CNT | 250 μm | 500 μm | 750 μm | 1000 μm |
| --- | --- | --- | --- | --- |
| 1:1 | 0.02 | 160.71 | 0.08 | 0.30 |
| 1:0.5 | 3.62 | 1.01 | 0.34 | 3.00 |
| 1:0.33 | 0.12 | 22.27 | 1014.27 | 72.48 |
| 1:0.25 | 58.99 | 305.42 | 124.99 | 6.90 |
| 1:0 | 6.45 | 199.36 | 191.40 | 0.07 |

**Table S4.** Performance comparation of recent reported piezoresistive pressure sensors based on paper and textile substrate.

| Materials | Sensitivity  (kPa^-1^) | Sensing range (kPa) | Minimum detection limit (Pa) | Response/recovery time | Ref.# |
| --- | --- | --- | --- | --- | --- |
| MXene-coated tissue paper | 509.5 (0.5-10 kPa),  179.4 (10-30 kPa),  53.7 (30-100 kPa) | 0.5-100 | 1 | N.A. | [20] |
| PEDOT:PSS@air-laid paper | 768.07 (0.02-15 kPa)  274.34 (15-100 kPa)  93.96 (100-250 kPa) | 0.02-250 | 20 | 120 ms  /80 ms | [23] |
| Graphene-paper | 17.2 (0-2 kPa),  0.1 (2-20 kPa) | 0-20 | N.A. | N.A. | [25] |
| AgNWs-coated tissue paper | 1.5 (0.03-30.2) | 0.03-30.2 | 30 | N.A. | [31] |
| Mxene/BC-paper | 51.14 (0-0.82 kPa),  2.62 (0.82-10.92) | 0-10.92 | N.A. | 99 ms  /93 ms | [42] |
| Mxene-based cotton fabric | 5.3 (0-1.3 kPa),  2.27 (1.3-10.25 kPa),  0.57 (10.25-40.73 kPa),  0.08 （40.73-160 kPa） | 0-160 | N.A. | 50 ms  /20 ms | [43] |
| CNT-coated cotton fabric. | 14.4 (0.002-3.5 kPa),  7.8 (3.5-15 kPa) | 0.002-15 | 2 | 18 ms  /24 ms | [44] |
| MX@SiNPs cotton textile | 12.23 (0-13 kPa),  2 (13-52 kPa),  8.46 (52-75 kPa) | 0-75 | 8.8 | N.A. | [45] |
| Organic/inorganic mixture-coated nonwoven | 3.67 (0-20 kPa),  10.03 (20-118 kPa) | 0-118 | N.A. | 135 ms  /100 ms | [46] |
| BP@PANI-based tactile | 5.57 (0.5-30 kPa),  0.154 (30-100 kPa) | 0-100 | 500 | 200 ms  /210 ms | [47] |
| **PEDOT:PSS@3DFC-CNT@filter paper** | **1014.27 (0.01-50 kPa),**  **203.12 (50-150 kPa),**  **37.49 (150-300 kPa)** | **0.01-300** | **＜10** | **105 ms**  **/45 ms** | **This work** |


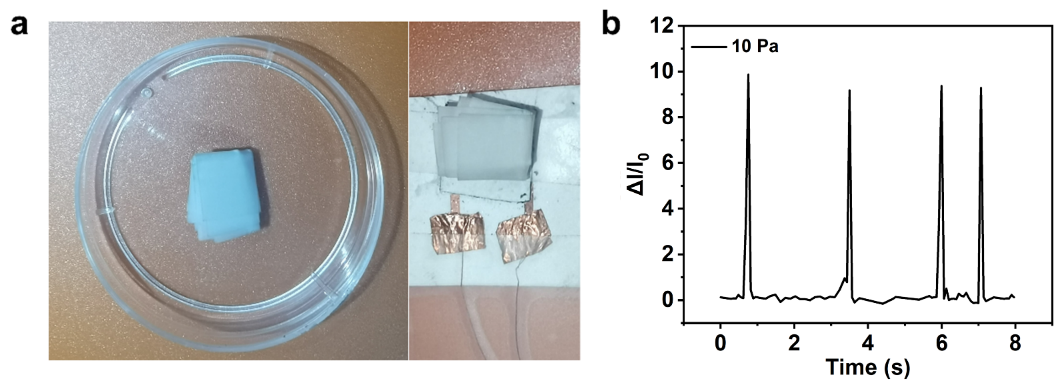


**Figure S9. (a)** Digital camera diagram for micro force measurement and (b)The current response during loading-unloading test at a pressure of 0.001 N.


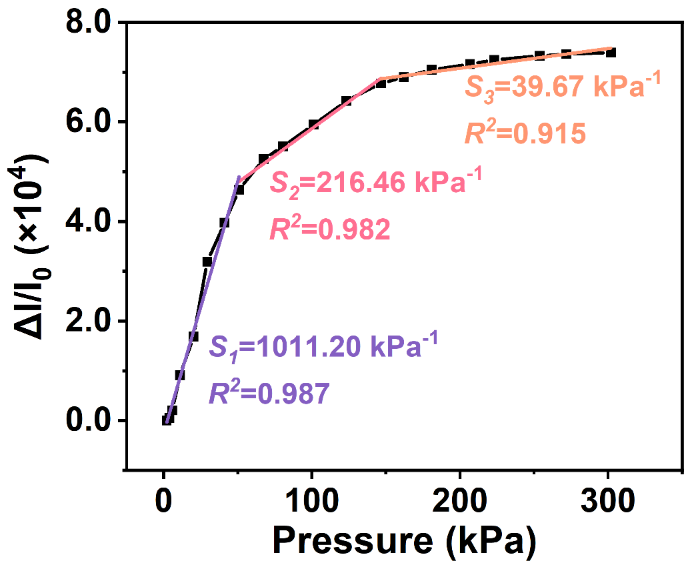


**Figure S10.** Sensitivity of the all-paper piezoresistive pressure sensor stored for 150 days.


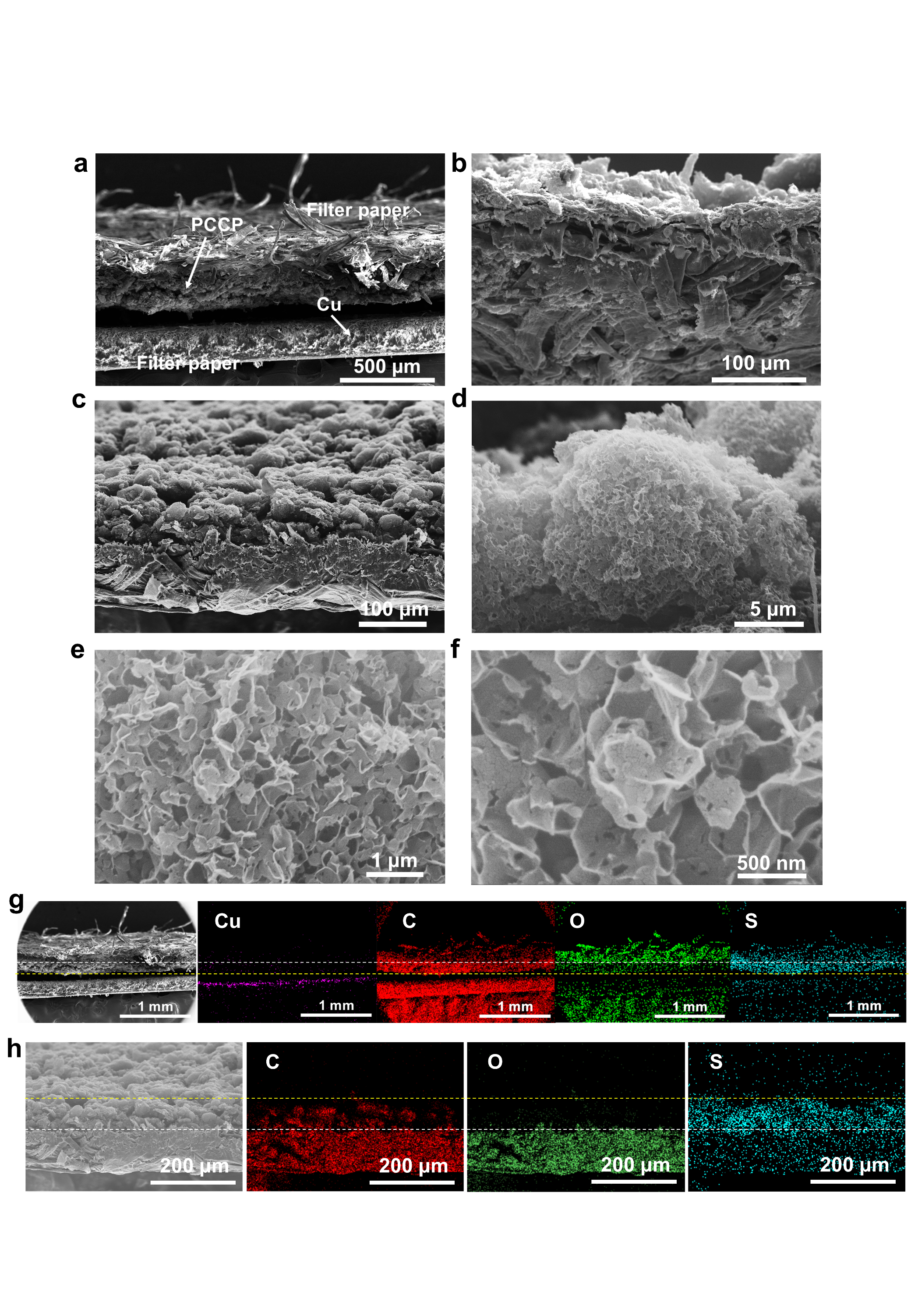


**Figure S11.** (a) Cross-sectional SEM image of the all-paper sensor, including the top and bottom electrodes and the encapsulation layer. (b), (c) Cross-sectional SEM image of PEDOT:PSS@3DFC-CNT film. (d)-(f) Magnified image of panel c. (g) Cross-sectional SEM image and EDX maps scanning of the all-printed sensors. (h) Cross-sectional SEM image and EDX maps scanning of PEDOT:PSS@3DFC-CNT on paper (top electrode).

In Figure S11a, the sensor exhibits a unique hierarchical structure. What’s more, PCCP exhibits a hemispherical protrusion as a whole, reflecting the micrometer scale structure possessed by PCCP (Figure S11b and Figure S11c). In Figures S11d, e, and f, at a larger magnification, the nanoscale pores of PCCP are clearly visible, providing structural support for the deformation of the sensor and also proving the nanostructure possessed by PCCP. These SEM images suggest that the composite materials contain surface microstructure/nanostructure and the internal porous nanostructure.


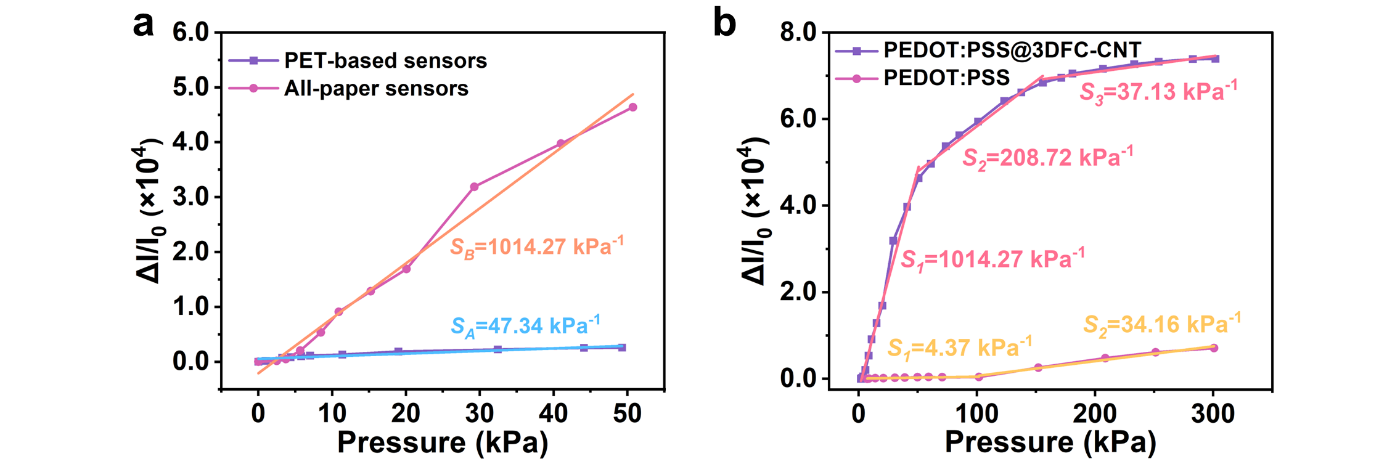


**Figure S12.** (a) The sensor sensitivity curves with different flexible substrates at the same 3DFC:CNT ratio and gap clearance of blade coating. (b) The sensor sensitivity curves with PEDOT:PSS@3DFC-CNT thin film electrodes and PEDOT:PSS thin film electrodes on paper.


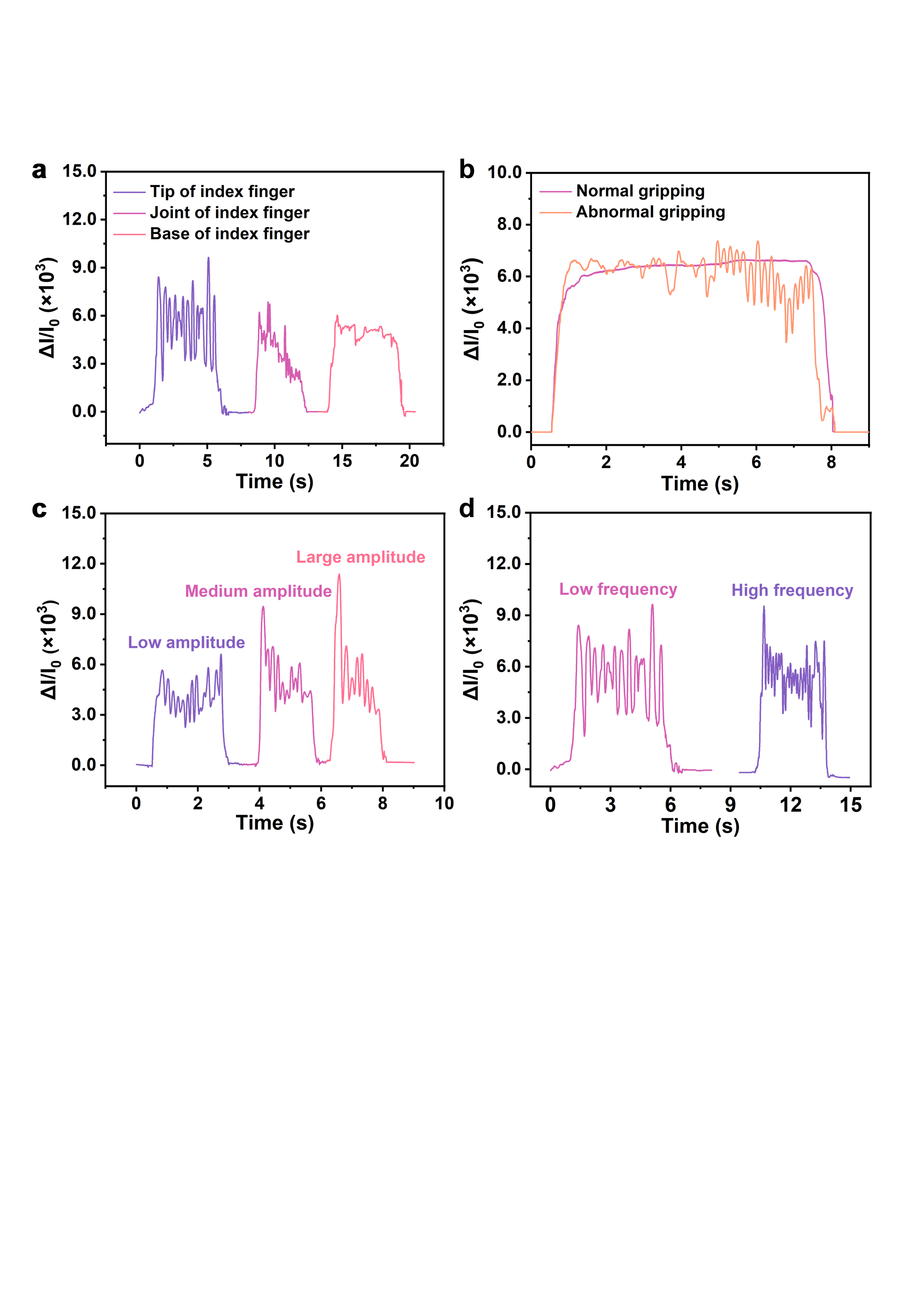


**Figure S13.** (a) Current response curves of the sensor at different jitter locations. (b) Current response curves of the sensor at the tip of the index finger under normal grip and abnormal bottle gripping (jitter). (c) Current response curves of the sensor at different jitter amplitudes. (d) Current response curves of the sensor at different jitter frequencies.

In Figure S13a, current response curves of the sensor array at different jitter locations has been tested. Representative current signals from index fingertips, index knuckles, and index finger bases are selected, clearly showing the jitter of various parts of the hand during hand grasping. Compared with normal grip, the current signal corresponding to the grasping posture under jitter has obvious signal differences (Figure S13b). In addition, the fingertip signal with the most significant vibration signal is selected for further exploration. At different jitter amplitudes (Figure S13c) and different jitter frequencies (Figure S13d), the sensing arrays exhibit distinct current signal characteristics. With different jitter amplitudes, as the amplitude of the signal from the sensing array increases, the current fluctuations become more pronounced. With different jitter frequencies, as the vibration frequency increases, the number of waveforms in the response current curve increases.
